# Complete Genome Sequence of *Bacillus velezensis* GQJK49, a Plant Growth-Promoting Rhizobacterium with Antifungal Activity

**DOI:** 10.1128/genomeA.00922-17

**Published:** 2017-08-31

**Authors:** Jinjin Ma, Hu Liu, Kai Liu, Chengqiang Wang, Yuhuan Li, Qihui Hou, Liangtong Yao, Yanru Cui, Tongrui Zhang, Haide Wang, Beibei Wang, Yun Wang, Ruofei Ge, Baochao Xu, Gan Yao, Wenfeng Xu, Lingchao Fan, Yanqin Ding, Binghai Du

**Affiliations:** a College of Life Sciences/Shandong Key Laboratory of Agricultural Microbiology/National Engineering Laboratory for Efficient Utilization of Soil and Fertilizer Resources, Shandong Agricultural University, Tai’an, China; b College of Resources and Environment, Shandong Agricultural University, Tai’an, China; c State Key Laboratory of Nutrition Resources Integrated Utilization, Linshu, China

## Abstract

*Bacillus velezensis* GQJK49 is a plant growth-promoting rhizobacterium with antifungal activity, which was isolated from *Lycium barbarum* L. rhizosphere. Here, we report the complete genome sequence of *B. velezensis* GQJK49. Twelve gene clusters related to its biosynthesis of secondary metabolites, including antifungal and antibacterial antibiotics, were predicted.

## GENOME ANNOUNCEMENT

*Bacillus velezensis* is widely used as a biocontrol strain (1–3). Chang et al. (4) reported that *B. velezensis* SSH100-10 produces iturin A against fungi. Phenol (4-chloro-3-methyl) (5) production of *B. velezensis* ZSY-1 can suppress* Alternaria solani* and *Botrytis cinerea*. Roh et al. (1) reported that *B. velezensis* exhibited activity against *Magnaporthe grisea*, *Rhizotonia solani*, *Botrytis cinerea*, *Phytophthora infestans*, and *Puccinia recondite*. In addition, *B. velezensis* promotes the growth of a variety of plants (6). *B. velezensis* GQJK49 was isolated from rhizosphere of *Lycium barbarum* L. in Ningxia, China. It has significant inhibition effects on *Fusarium solani*, which causes root rot of *Lycium barbarum* L.

Complete genome sequencing of *B. velezensis* GQJK49 was performed using a PacBio (8- to 10-kb) platform. A total of 111,774 reads, containing 986,581,491 bp, were generated. The largest reads contained 46,246 bp, and the average length of reads was 8,826.6 bp. The genome coverage was 251×. The *de novo* assembly of reads produced by PacBio was performed using Canu v1.3 (7). Glimmer 3.02 (8) (http://ccb.jhu.edu/software/glimmer/index.shtml) was used to annotate the complete genome of *B. velezensis* GQJK49. The carbohydrate active enzyme analyses of the genome were performed by use of the Carbohydrate Active enZYmes database (CAZy) (9) version 20141020 (http://www.cazy.org/). Prophages were predicted with PHAST (10). Secondary metabolites were predicted by antiSMASH (11) version 3.0.5 (http://antismash.secondarymetabolites.org/).

The complete genome of *B. velezensis* GQJK49 comprised 3,929,760 bp, with a GC content of 46.50%. A total of 3,921 genes, including 86 tRNA genes and 27 rRNA genes, were annotated by Glimmer 3.02. The genome has 3,677 coding genes and the length of sequences was 3,506,193 bp. The gene density was 1.030 genes per kb. GC content in the gene region was 47.2%. We found that 136 genes were related to carbohydrate enzymes, including 44 genes involved in glycoside hydrolases (GHs), 38 genes coding glycoside transferases (GTs), 30 genes coding carbohydrate esterases (CEs), and 24 genes related to carbohydrate-binding modules (CBMs), auxiliary activities (AAs), or polysaccharide lyases (PLs). A prophage of about 34 kb was predicted by PHAST. There were 12 gene clusters related to antimicrobial activity. Six of them presented high similarity with the biosynthesis gene clusters of relevant secondary metabolism. Two transAT polyketide synthase-nonribosomal peptide synthetase (TATPKS-NRPS)-type clusters (BAGQ_1558 to BAGQ_1605 and BAGQ_2358 to BAGQ_2413) showed similarity with the biosynthetic gene clusters of macrolactin and difficidin, respectively. Two gene clusters (BAGQ_1833 to BAGQ_1882 and BAGQ_1959 to BAGQ_2031), which belonged to the transAT TATPKS-NRPS type, were related to bacillaene and fengycin, respectively. One gene cluster (BAGQ_3143 to BAGQ_3210) belonged to NRPS-bacteriocin, which was similar to the biosynthetic gene cluster of bacteriocin. One gene cluster (BAGQ_3790 to BAGQ_3835) was related to bacilysin biosynthesis. The other gene clusters may be related to the production of new antimicrobial substances. The complete genome sequence of *B. velezensis* GQJK49 will be helpful in the study of its mechanisms for biocontrol and plant growth promotion and will facilitate the expansion of the scope of application of this strain in agriculture.

### Accession number(s).

The chromosome sequence of *B. velezensis* GQJK49 has been deposited at GenBank under accession number CP021495.
